# Parameter Estimation of Platelets Deposition: Approximate Bayesian Computation With High Performance Computing

**DOI:** 10.3389/fphys.2018.01128

**Published:** 2018-08-20

**Authors:** Ritabrata Dutta, Bastien Chopard, Jonas Lätt, Frank Dubois, Karim Zouaoui Boudjeltia, Antonietta Mira

**Affiliations:** ^1^Institute of Computational Science, Università della Svizzera italiana, Lugano, Switzerland; ^2^Computer Science Department, University of Geneva, Geneva, Switzerland; ^3^Microgravity Research Centre, Université libre de Bruxelles (ULB), Brussels, Belgium; ^4^Laboratory of Experimental Medicine (ULB 222 Unit), Université Libre de Bruxelles and CHU de Charleroi, Brussels, Belgium; ^5^Department of Science and High Technology, Università degli Studi dell'Insubria, Varese, Italy

**Keywords:** platelet deposition, numerical model, Bayesian inference, approximate Bayesian computation, high performance computing

## Abstract

Cardio/cerebrovascular diseases (CVD) have become one of the major health issue in our societies. Recent studies show the existing clinical tests to detect CVD are ineffectual as they do not consider different stages of platelet activation or the molecular dynamics involved in platelet interactions. Further they are also incapable to consider inter-individual variability. A physical description of platelets deposition was introduced recently in Chopard et al. (2017), by integrating fundamental understandings of how platelets interact in a numerical model, parameterized by five parameters. These parameters specify the deposition process and are relevant for a biomedical understanding of the phenomena. One of the main intuition is that these parameters are precisely the information needed for a pathological test identifying CVD captured and that they capture the inter-individual variability. Following this intuition, here we devise a Bayesian inferential scheme for estimation of these parameters, using experimental observations, at different time intervals, on the average size of the aggregation clusters, their number per mm^2^, the number of platelets, and the ones activated per μℓ still in suspension. As the likelihood function of the numerical model is intractable due to the complex stochastic nature of the model, we use a likelihood-free inference scheme approximate Bayesian computation (ABC) to calibrate the parameters in a data-driven manner. As ABC requires the generation of many pseudo-data by expensive simulation runs, we use a high performance computing (HPC) framework for ABC to make the inference possible for this model. We consider a collective dataset of seven volunteers and use this inference scheme to get an approximate posterior distribution and the Bayes estimate of these five parameters. The mean posterior prediction of platelet deposition pattern matches the experimental dataset closely with a tight posterior prediction error margin, justifying our main intuition and providing a methodology to infer these parameters given patient data. The present approach can be used to build a new generation of personalized platelet functionality tests for CVD detection, using numerical modeling of platelet deposition, Bayesian uncertainty quantification, and High performance computing.

## 1. Introduction

Blood platelets play a major role in the complex process of blood coagulation, involving adhesion, aggregation, and spreading on the vascular wall to stop a hemorrhage while avoiding the vessel occlusion. Platelets also play a key role in the occurrence of cardio/cerebro-vascular accidents that constitute a major health issue in our societies. In 2015, Cardiovascular diseases (CVD), including disorders of the heart and blood vessels, were the first cause of mortality worldwide, causing 31% of deaths (Organization, 2015). Antiplatelet therapy generally reduces complications in patients undergoing arterial intervention (Mehta et al., 2001; Steinhubl et al., 2002). However, the individual response to dual antiplatelet therapy is not uniform and consistent studies reported that even under platelets therapy there were recurrences of atherothrombotic events (Matetzky et al., 2004; Gurbel et al., 2005; Geisler et al., 2006; Hochholzer et al., 2006; Marcucci et al., 2009; Price et al., 2008; Sibbing et al., 2009). In most cases, a standard posology is prescribed to patients, which does not take into account the inter-individual variability linked to the absorption or the effectiveness of these molecules. This was supported by a recent study (Koltai et al., 2017), reporting the high patient-dependency of the response of the antithrombotic drugs. We should also note that the evaluation of the response to a treatment by the existing tests is test-dependent.

Nowadays, platelet function testing is performed either as an attempt to monitor the efficacy of anti-platelet drugs or to determine the cause of abnormal bleeding or pro-thrombotic status. The most common method consists of using an optical aggregometer that measures the transmittance of light passing through plasma rich in platelets (PRP) or whole blood (Born and Cross, 1963; Harrison, 2009), to evaluate how platelets tend to aggregate. Other aggregometers determine the amount of aggregated platelets by electric impedance (Velik-Salchner et al., 2008) or luminescence. In specific contexts, flow cytometry (Michelson et al., 2002) is also used to assess platelet reactivity (VASP test; Bonello et al., 2009). Determination of platelet functions using these different existing techniques in patients undergoing coronary stent implantation have been evaluated in Breet et al. (2010), which shows the correlation between the clinical biological measures and the occurrence of a cardiovascular event was null for half of the techniques and rather modest for others. This may be due to the fact that no current test allows the analysis of the different stages of platelet activation or the prediction of the *in vivo* behavior of those platelets (Picker, 2011; Koltai et al., 2017). It is well-known that the phenomenon of platelet margination (the process of bringing platelets to the vascular wall) is dependent on the number and shape of red blood cells and their flow (Piagnerelli et al., 2007), creating different pathologies for different diseases (e.g., diabetes, End Renal Kidney Disease, hypertension, sepsis). Further, platelet margination is also known to be influenced by the aspect ratio of surrogate platelet particles (Reasor et al., 2013). Although there is a lot of data reported by recent research works (Maxwell et al., 2007) on the molecules involved in platelet interactions, these studies indicate that there is a lack of knowledge on some fundamental mechanisms that should be revealed by new experiments.

*Hence, the challenge is to find parameters connecting the dynamic processes of adhesion and aggregation of platelets to the data collected from the individual patients*. Recently, by combining digital holography microscopy (DHM) and mathematical modeling, (Chopard et al., 2015; Boudejltia et al., 2015; Chopard et al., 2017) provided a physical description of the adhesion and aggregation of platelets in the Impact-R device. A numerical model is developed that quantitatively describes how platelets in a shear flow adhere and aggregate on a deposition surface. This is the first innovation in understanding the molecular dynamics involved in platelet interactions. Five parameters specify the deposition process and are relevant for a biomedical understanding of the phenomena. One of the main intuition is that the values of these parameters (e.g., adhesion and aggregation rates) are precisely the information needed to assess various possible pathological situations and quantify their severity regarding CVD. Further, it was shown in Chopard et al. (2017) that, by hand-tuning the parameters of the mathematical model, the deposition patterns observed for a set of healthy volunteers in the Impact-R can be reproduced.

*Assuming that these parameters can determine the severity of CVD, how do we estimate the adhesion and aggregation rates of given patients by a clinical test?* The determination of these adhesion and aggregation rates by hand-tuning is clearly not a solution as we need to search the high-dimensional parameter space of the mathematical model, which becomes extremely expensive and time consuming. We further notice, this has to be repeated for each patient and thus requires a powerful numerical approach. In this work, we resolve the question of estimating the parameters using Bayesian uncertainty quantification. Due to a complex stochastic nature, the numerical model for platelet deposition does not have a tractable likelihood function. We use Approximate Bayesian Computation (ABC), a likelihood-free inference scheme, with an optimal application of HPC (Dutta et al., 2017a) to provide a Bayesian way to estimate adhesion and aggregation rates given the deposition patterns observed in the Impact-R of platelets collected from a patient. Obviously, the clinical applicability of the proposed technique to provide a new platelet function test remains to be explored, but the numerical model (Chopard et al., 2017) and the proposed inference scheme here, bring the technical elements together to build a new class of medical tests.

In section 2 we introduce the necessary background knowledge about the platelet deposition model, whereas section 3 recalls the concept of Bayesian inference and introduces the HPC framework of ABC used in this study. Then we illustrate the results of the parameter determination for platelet deposition model using ABC methodology, collectively for seven patients in section 4. Clearly, the same methodology can be used to determine the parameter values for each individual patients in a similar manner for a CVD clinical test. Finally, in section 5 we conclude the paper and discuss its impact from a biomedical perspective.

## 2. Background and scientific relevance

The Impact-R (Shenkman et al., 2008) is a well-known platelet function analyzer. It is a cylindrical device filled in with whole blood from a donor. Its lower end is a fixed disk, serving as a deposition surface, on which platelets adhere and aggregate. The upper end of the Impact-R cylinder is a rotating cone, creating an adjustable shear rate in the blood. Due to this shear rate, platelets move toward the deposition surface, where they adhere or aggregate. Platelets aggregate next to already deposited platelets, or on top of them, thus forming clusters whose size increase with time. This deposition process has been successfully described with a mathematical model in Chopard et al. (2015); Chopard et al. (2017).

The numerical model (coined M in what follows) requires five parameters that specify the deposition process and are relevant for a bio-medical understanding of the phenomena. In short, the blood sample in the Impact-R device contains an initial number ℕ_*platelet*_(0) of non-activated platelets per μℓ and a number ℕ_*act*−*platelet*_(0) of pre-activated platelets per μℓ. Initially both type of platelets are supposed to be uniformly distributed within the blood. Due to the process known as shear-induced diffusion, platelets hit the deposition surface. Upon such an event, an activated platelets will adhere with a probability that depends on its adhesion rate, *pAd*, that we would like to determine. Platelets that have adhered on the surface are the seed of a cluster that can grow due to the aggregation of the other platelets reaching the deposition surface. We denote with *pAg* the rate at which new platelets will deposit next to an existing cluster. We also introduce *pT* the rate at which platelets deposit on top of an existing cluster. An important observation made in Chopard et al. (2015); Chopard et al. (2017) is that albumin, which is abundant in blood, compete with platelet for deposition. This observation is compatible with results reported in different experimental settings (Sharma et al., 1981; Remuzzi and Boccardo, 1993; Fontaine et al., 2009). As a consequence, the number of aggregation clusters and their size tends to saturate as time goes on, even though there are still a large number of platelets in suspension in the blood.

**Figure 1:**
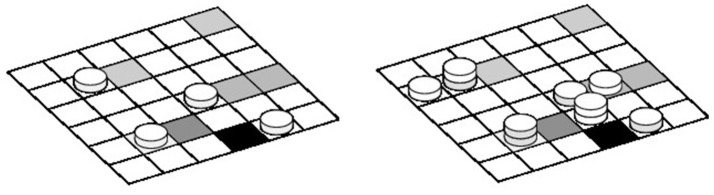
Sketch of the deposition substrate, discretized in cells of area equal to the surface of a platelet.

To describe this process in the model, two extra parameters, *pF*, the deposition rate of albumin, and *aT*, a factor that accounts for the decrease of platelets adhesion and aggregation on locations where albumin has already deposited, were introduced. The numerical model is described in full detail in Chopard et al. (2015); Chopard et al. (2017). Here we simply repeat the main elements. Due to the mixing in the horizontal direction, it was assumed that the activated platelets (AP), non-activated platelets (NAP) and albumin (Al) in the bulk can be described by a 1D diffusion equation along the vertical axis *z*

(1) $$\begin{array}{r} \partial_{t}\rho =D\partial_{z}^{2}\rho \;J=-D\text{grad}\rho \end{array}$$

where ρ is the density of either AP, NAP or Al, *J* and *D* are correspondingly the flux of particles and the shear induced diffusion. Upon reaching a boundary layer above the deposition substrate, adhesion and aggregation will take place according to

(2) $$\begin{array}{r} Ṅ=-J\left(0,t\right)\Delta S-p_{d}N\left(t\right) \end{array}$$

where *N* is the number of particles in the boundary layer, Δ*S* a surface element on the deposition surface, and *p*_*d*_ is the deposition rate, which evolves during time and varies across the substrate, according to the deposition history. For the deposition process, particles are considered as discrete entities that can attach to any position of the grid representing the deposition surface, as sketched in Figure 1. In this figure, the gray levels illustrate the density of albumin already deposited in each cell. The picture also illustrates the adhesion, aggregation, and vertical deposition along the *z*-axis. On the left panel, activated platelets (gray side disks) deposit first. Then in the second panel, non-activated platelets (white side disks) aggregate next to an already formed cluster. Both pre-activated and non-activated platelets can deposit on top of an existing cluster.

The deposition rules are the following. An albumin that reaches the substrate at time *t* deposits with a probability *P*(*t*) which depends on the local density ρ_*al*_(*t*) of already deposited Al. We assume that *P* is proportional to the remaining free space in the cell,

(3) $$P(t)=pf(\rho_{max}-\rho_{al}(t)),$$

where *pF* is a parameter and ρ_*max*_ is determined by the constraint that at most 100,000 albumin particles can fit in a deposition cell of area Δ*S* = 5 (μ*m*)^2^, corresponding to the size of a deposited platelet (obtained as the smallest variation of cluster area observed with the microscope).

An activated platelet that hits a platelet-free cell deposits with a probability *Q*, where *Q* decreases as the local concentration ρ_*al*_ of albumin increases. We assumed that

(4) $$\begin{array}{r} Q=pAd\exp\left(-aT\rho_{al}\right), \end{array}$$

where *pAd* and *aT* are parameters. This expression can be justified by the fact that a platelet needs more free space than an albumin to attach to the substrate, due to their size difference. In other words, the probability of having enough space for a platelet, decreases roughly exponentially with the density of albumin in the substrate. This can be validated with a simple deposition model on a grid, where small and large objects compete for deposition.

Once an activated platelet has deposited, it is the seed of a new cluster that grows further due to the aggregation of further platelets. In our model, AP and NAP can deposit next to already deposited platelets. From the above discussion, the aggregation probability *R* is assumed to be

(5) $$R=p_{Ag}\exp\left(-a_{T}\rho_{al}\right),$$

with *pAg* another parameter.

The above deposition probabilities can also be expressed as deposition rate over the given simulation time step Δ*t* = 0.01*s* (see Chopard et al., 2017 for details), hence giving a way to couple the diffusion Equation (1) with the 2D discrete deposition process sketched in Figure 1. Particles that did not deposit at time *t* are re-injected in the bulk and contribute to boundary condition of Equation (1) at *z* = 0.

To the best of our knowledge, except for Chopard et al. (2015); Chopard et al. (2017) there is no model in the literature that describes quantitatively the proposed *in-vitro* experiment. The closest approach is that of Affeld et al. (2013) but albumin is not included, and the role of pre-activated and non-activated platelets is not differentiated. Also, we are not aware of any other study than ours that reports both the amount of platelets in suspension as a function of time and those on the deposition surface.

The validity of the proposed numerical model has been explored in detail in Chopard et al. (2017). This validation is based on the fact that the model, using hand-tuned parameters can reproduce the time-dependent experimental observations very well. We refer the readers to Chopard et al. (2017) for a complete discussion. Here we briefly recall the main elements that demonstrate the excellent agreement of the model and the simulations. We reproduce Figure 2 from Chopard et al. (2017), showing the visual similarity between the actual and simulated deposition pattern. In the validation study, the evolution of the number of clusters, their average size and the numbers of pre-activated and non-activated platelets still in suspension matched quantitatively with the experimental measurements at times 20, 60, 120, and 300 s. In addition, a very good agreement between the simulated deposition pattern and the experiment was also found by comparing the distributions of the areas and volumes of the aggregates.

**Figure 2:**
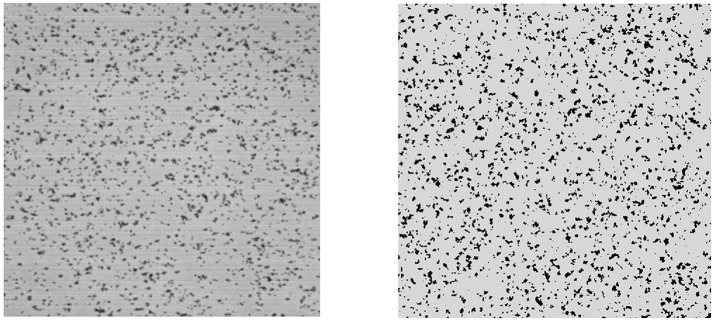
The deposition surface of the Impact-R device after 300 s **(Left)** and the corresponding results of the deposition in the mathematical model **(Right)**. Black dots represent the deposited platelets that are grouped in clusters.

**Figure 6:**
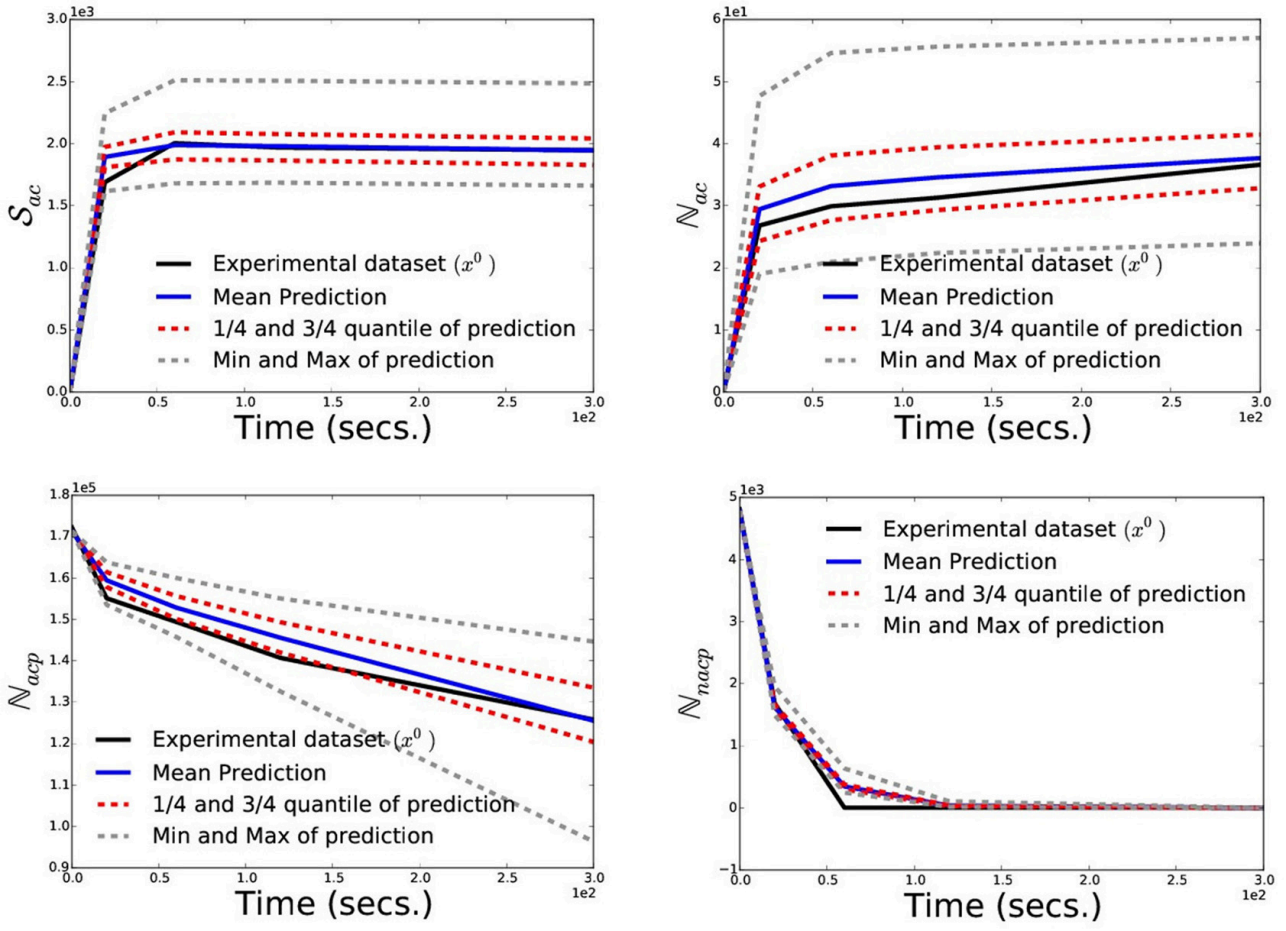
Posterior Prediction Check: To validate the numerical model of the platelet deposition and the inference scheme we perform a posterior prediction check by simulating 100 datasets, each using a different parameter sample drawn from the posterior distribution. Here, we plot the experimental dataset (black-solid) used for inference, mean predicted dataset (blue-solid), 1/4-th and 3/4-th quantile (red-dashed), minimum and maximum (gray-dashed) of the predicted datasets at each timepoints.

To be noticed, the validation reported in Chopard et al. (2017) was done using manually estimated parameters. As the main goal of this research is to propose an inference scheme to learn the parameters in a data-driven manner, a validation for the model and the inference scheme is reported in Figure 6 below, using the inferred posterior distribution which also includes a quantification of prediction error.

For the purpose of the present study, the model M is parametrized in terms of the five quantities introduced above, namely the adhesion rate *pAd*, the aggregation rates *pAg* and *pT*, the deposition rate of albumin *pF*, and the attenuation factor *aT*. Some additional parameters of the model, specifically, the shear-induced diffusion coefficient and the thickness of the boundary layer (Chopard et al., 2017), are assumed here to be known. Collectively, we define

$$\theta =\left(p_{Ag},p_{Ad},p_{T},a_{T}\right).$$

If the initial values for ℕ_*platelet*_(0) and ℕ_*act*−*platelet*_(0), as well as the concentration of albumin are known from the experiment, we can forward simulate the deposition of platelets over time using model M for the given values of these parameters **θ** = θ^*^:

(6) $$\begin{array}{l} M\left[\theta =\theta^{*}\right]\to \{\left(S_{agg-clust}\left(t\right),{\mathbb{N}}_{platelet}\left(t\right),{\mathbb{N}}_{act-platelet}\left(t\right)\right),\\ \;t=0,\ldots ,T\}. \end{array}$$

where $S_{agg-clust}\left(t\right),{\mathbb{N}}_{agg-clust}\left(t\right),{\mathbb{N}}_{platelet}\left(t\right)$ , and ℕ_*act*−*platelet*_(*t*) are correspondingly average size of the aggregation clusters, their number per mm^2^, the number of non-activated and pre-activated platelets per μℓ still in suspension at time *t*.

The Impact-R experiments have been repeated with the whole blood obtained from seven donors and the observations were made at time, 0 , 20 , 60 , 120, and 300 s. At these five time points, $\left[Sagg-clust\left(t\right),{\mathbb{N}}_{agg-clust}\left(t\right),{\mathbb{N}}_{platelet}\left(t\right),{\mathbb{N}}_{act-platelet}\left(t\right)\right]$ are measured. Let us call the observed dataset collected through experiment as,

(7) $$\begin{array}{l} x^{0}\equiv \{\left(S_{agg-clust}^{0}\left(t\right),{\mathbb{N}}_{platelet}^{0},(t){\mathbb{N}}_{act-platelet}^{0}\left(t\right)\right):\\ \;t=0\;s.,\ldots 300\;s.\} \end{array}$$

By comparing the number and size of the deposition aggregates obtained from the *in-vitro* experiments with the computational results obtained by forward simulation from the numerical model (see Figure 2 for an illustration), the model parameters were manually calibrated by a trial and error procedure in Chopard et al. (2017). Due to the complex nature of the model and high-dimensional parameter space, this manual determination of the parameter values are subjective and time consuming.

However, if the parameters of the model could be learned more rigorously with an automated data-driven methodology, we could immensely improve the performance of these models and bring this scheme as a new clinical test for platelet functions. To this aim, here we propose to use ABC for Bayesian inference of the parameters. As a result of Bayesian inference to this context, not only we can automatically and efficiently estimate the model parameters, but we can also perform parameter uncertainty quantification in a statistically sound manner, and determine if the provided solution is unique.

## 3. Bayesian inference

We can quantify the uncertainty of the unknown parameter **θ** by a posterior distribution *p*(**θ**|***x***) given the observed dataset ***x*** = ***x*^0^**. A posterior distribution is obtained, by Bayes' Theorem as,

(8) $$\begin{array}{r} p\left(\theta |x\right)=\frac{\pi \left(\theta \right)p\left(x|\theta \right)}{m\left(x\right)}, \end{array}$$

where π(**θ**), *p*(*x*|**θ**) and *m*(***x***) = ∫π(**θ**)*p*(***x***|**θ**)*d****θ*** are correspondingly the prior distribution on the parameter **θ**, the likelihood function, and the marginal likelihood. The prior distribution π(**θ**) ensures a way to leverage the learning of parameters with prior knowledge, which is commonly known due to the availability of medical knowledge regarding cardio-vascular diseases. If the likelihood function can be evaluated, at least up to a normalizing constant, then the posterior distribution can be approximated by drawing a sample of parameter values from the posterior distribution using (Markov chain) Monte Carlo sampling schemes (Robert and Casella, 2005). For the simulator-based models considered in section 2, the likelihood function is difficult to compute as it requires solving a very high dimensional integral. In next subsection 3.1, we illustrate ABC to perform Bayesian Inference for models where the analytical form of the likelihood function is not available in closed form or not feasible to compute.

### 3.1. Approximate bayesian computation

ABC allows us to draw samples from the approximate posterior distribution of parameters of the simulator-based models in absence of likelihood function, hence to perform approximate statistical inference (e.g., point estimation, hypothesis testing, model selection etc.) in a data-driven manner. In a fundamental Rejection ABC scheme, we simulate from the model M(θ) a synthetic dataset *x*sim for a parameter value **θ** and measure the closeness between ***x*^sim^** and ***x*^0^** using a pre-defined discrepancy function *d*(***x*^sim^**, ***x*^0^**). Based on this discrepancy measure, ABC accepts the parameter value θ when *d*(***x*^sim^**, ***x*^0^**) is less than a pre-specified threshold value ϵ.

As the Rejection ABC scheme is computationally inefficient, to explore the parameter space in an efficient manner, there exists a large group of ABC algorithms (Marin et al., 2012). As pointed in (Dutta et al., 2017a), these ABC algorithms, consist of four fundamental steps:

1. (Re-)sample a set of parameters **θ** either from the prior distribution or from an already existing set of parameter samples;
2. For each of the sample from the whole set or a subset, perturb it using the perturbation kernel, accept the perturbed sample based on a decision rule governed by a threshold or repeat the whole second step;
3. For each parameter sample calculate its weight;
4. Normalize the weights, calculate a co-variance matrix and adaptively re-compute the threshold for the decision rule.

These four steps are repeated until the weighted set of parameters, interpreted as the approximate posterior distribution, is “sufficiently close” to the true posterior distribution. The steps (1) and (4) are usually quite fast, compared to steps (2) and (3), which are the computationally expensive parts.

These ABC algorithms can be generally classified into two groups based on the decision rule in step (2). In the first group, we simulate ***x*^sim^** using the perturbed parameter and accept it if *d*(***x*^sim^**, ***x*^0^**) < ϵ, an adaptively chosen threshold. Otherwise we continue until we get an accepted perturbed parameter. For the second group of algorithms, we do not have this “explicit acceptance” step but rather a probabilistic one. Here we accept the perturbed parameter with a probability that depends on ϵ; if it is not accepted, we keep the present value of the parameter. The algorithms belonging to the “explicit acceptance” group are RejectionABC (Tavaré et al., 1997) and PMCABC (Beaumont, 2010), whereas the algorithms in the “probabilistic acceptance” group are SMCABC (Del Moral et al., 2012), RSMCABC (Drovandi and Pettitt, 2011), APMCABC Lenormand et al. (2013), SABC (Albert et al., 2015), and ABCsubsim Chiachio et al. (2014). For an “explicit acceptance” to occur, it may take different amounts of time for different perturbed parameters (more repeated steps are needed if the proposed parameter value is distant from the true parameter value). Hence the first group of algorithms are inherently imbalanced. We notice that an ABC algorithm with “probabilistic acceptance” do not have the similar issue of imbalance as a probabilistic acceptance step takes approximately the same amount of time for each parameter.

The generation of ***x*^sim^** from the model, for a given parameter value, usually takes up huge amounts of computational resources (e.g., 10 min for the platelets deposition model in this paper). Hence, we want to choose an algorithm with faster convergence to the posterior distribution with minimal number of required forward simulations. For this work we choose Simulated Annealing ABC (SABC) which uses a probabilistic decision rule in Step (2) and needs minimal number of forward simulation than other algorithms as shown in Albert et al. (2015). As all tasks of SABC in Step (2) can be run independently, in our recent work Dutta et al., 2017a, we have adapted SABC for HPC environment. Our implementation is available in Python package ABCpy and shows a linear scalability.

We further note that the parallelization schemes in ABCpy were primarily meant for inferring parameters from models, for which forward simulation takes almost equal time for any values of **θ**. Due to the complex stochastic nature of the numerical model, forward simulation time for different values of θ, can be quite variable. To solve this imbalance in the forward simulation, additionally to the imbalance reported for ABC algorithms, we use a new dynamic allocation scheme for MPI developed in Dutta et al. (2017b).

### 3.2. Dynamic allocation for MPI

Here we briefly discuss how a dynamic allocation strategy for map-reduce provides better balancing of ABC algorithms compared to a straightforward allocation approach.

**Figure 3:**
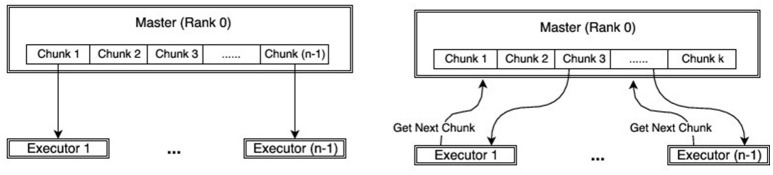
Comparison of work-flow between MPI **(Left)** and dynamic-MPI backend **(Right)**.

In the straightforward approach, the allocation scheme initially distributes *m* tasks to *n* executors, sends the map function to each executor, which in turn applies the map function, one after the other, to its *m*/*n* map tasks. This approach is visualized in Figure 3, where a chunk represents the set of *m*/*n* map tasks. For example, if we want to draw 10, 000 samples from the posterior distribution and we have *n* = 100 cores available, at each step of SABC we create groups of 100 parameters and each group is assigned to one individual core.

On the other hand, the dynamic allocation scheme initially distributes *k* < *m* tasks to the *k* executors, sends the map function to each executor, which in turn applies it to the single task available. In contrast to the straightforward allocation, the executor requests a new map task as soon as the old one is terminated. This clearly results in a better balance of the work. The dynamic allocation strategy is an implementation of the famous greedy algorithm for job-shop scheduling, which can be shown to have an overall processing time (makespan) up to twice as better than the best makespan (Graham, 1966).

**Figure 4:**
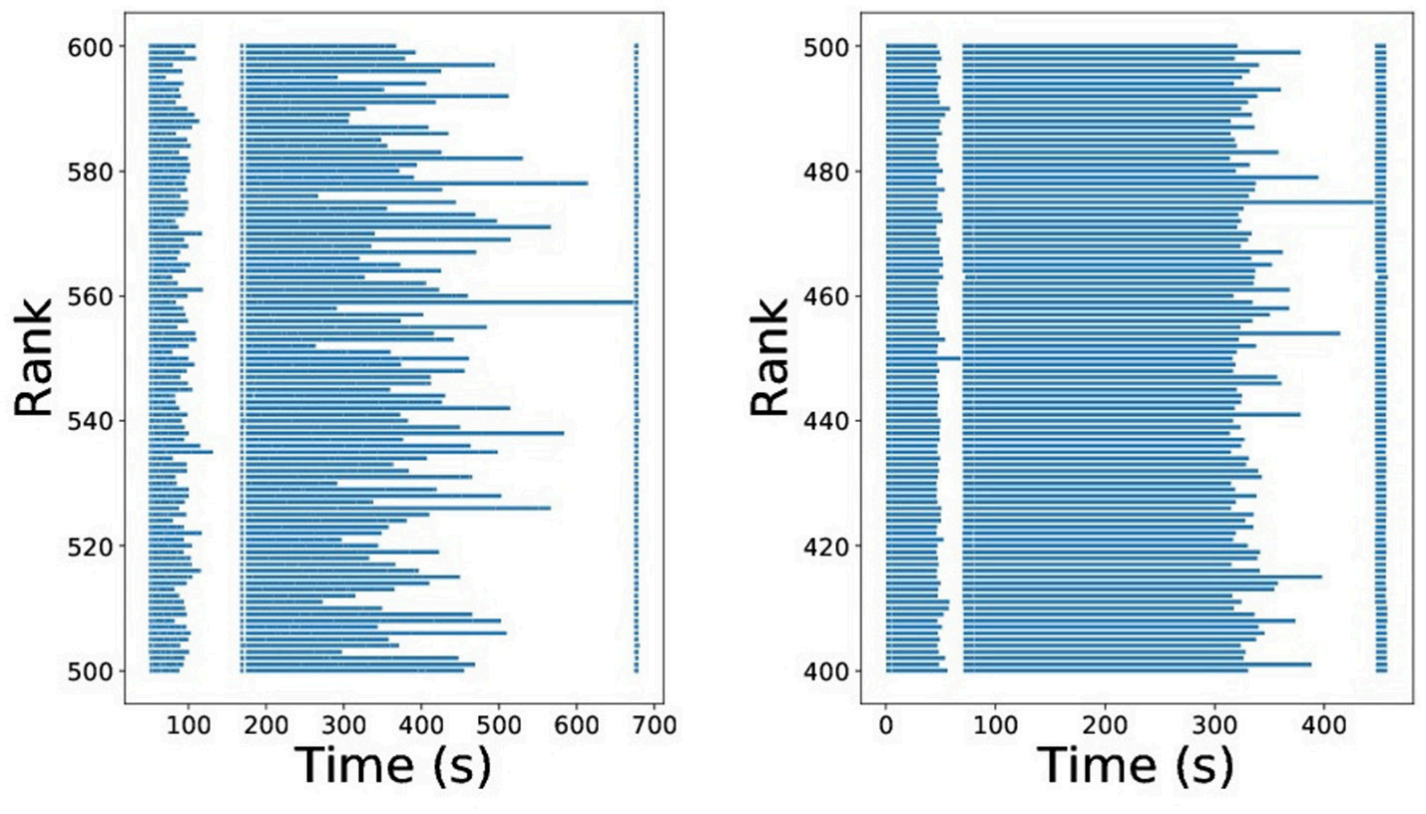
Imbalance of ABC algorithms using MPI(straight-forward) **(Left)** and MPI(dynamic-allocation) backend **(Right)**.

This approach is illustrated in Figure 3, reproduced from Dutta et al. (2017b). The unbalanced behavior is apparent if we visualize the run time of the individual map tasks on each executor. In Figure 4, the individual map tasks processing time is shown for an ABC algorithm performing inference on a weather prediction model, reported in Dutta et al. (2017b). Each row corresponds to an executor (or rank) and each bar corresponds to the total time spent on all tasks assigned to the respective rank (row) for one map call. For the straightforward allocation strategy, one can easily verify that most of the ranks finish their map tasks in half the time of the slowest rank. This clearly leads to large inefficiencies. Conversely, using the dynamic allocation strategy, the work is more evenly distributed across the ranks. The unbalancedness is not a problem that can be overcome easily by adding resources, rather speed-up and efficiency can drop drastically compared to the dynamic allocation strategy with increasing number of executors. For a detailed description and comparison, we direct readers to Dutta et al. (2017b).

### 3.3. Posterior inference

Using SABC within HPC framework implemented in ABCpy (Dutta et al., 2017a), we draw *Z* = 5000 samples approximating the posterior distribution *p*(**θ**|***x*^0^**), while keeping all the tuning parameters for the SABC fixed at the default values suggested in ABCpy package, except the number of steps and the acceptance rate cutoff, which was chosen respectively as 30 and 1*e*^−4^. The parallelized SABC algorithm, using HPC makes it possible to perform the computation in 5 h [using 140 nodes with 36-core of Piz Daint Cray architecture (Intel Broadwell + NVidia TESLA P100)], which would have been impossible by a sequential algorithm. To perform SABC for the platelets deposition model, the summary statistics extracted from the dataset, discrepancy measure between the summary statistics, prior distribution of parameters, and perturbation Kernel to explore the parameter space for inference are described next.

#### Summary statistics

Given a dataset, $x\equiv \left\{\left(S_{agg-clust}\left(t\right),{\mathbb{N}}_{agg-clust}\left(t\right),{\mathbb{N}}_{platelet}\left(t\right),{\mathbb{N}}_{act-platelet}\left(t\right)\right)\right\}(t)):t=0s.,\ldots ,300s.\}$ ℕ_*act*−*platelet*_(*t*)):*t* = 0*s*., …, 300*s*.}, we compute an array of summary statistics.

$$\begin{array}{l} F:\;x\to \left(\mu ,\sigma ,ac,c,cc\right) \end{array}$$

defined as following,

- **μ** = (μ_1_, μ_2_, μ_3_, μ_4_), mean over time.
- **σ** = (σ_1_, σ_2_, σ_3_, σ_4_), variance over time.
- ***ac*** = (*ac*_1_, *ac*_2_, *ac*_3_, *ac*_4_), auto-correlation with lag 1.
- ***c*** = (*c*_1_, *c*_2_, *c*_3_, *c*_4_, *c*_5_, *c*_6_), correlation between different pairs of variables over time.
- ***cc*** = (*cc*_1_, *cc*_2_, *cc*_3_, *cc*_4_, *cc*_5_, *cc*_6_), cross-correlation with lag 1 between different pairs of variables over time.

The summary statistics, described above, are chosen to capture the *mean* values, *variances*, and the *intra-* and *inter-* dependence of different variables of the time-series over time.

#### Discrepancy measure

Assuming the above summary statistics contain the most *essential* information about the likelihood function of the simulator-based model, we compute Bhattacharya-coefficient (Bhattachayya, 1943) for each of the variables present in the time-series using their mean and variance and Euclidean distances between different *inter-* and *intra-* correlations computed over time. Finally we take a mean of these discrepancies, such that, in the final discrepancy measure discrepancy between each of the summaries are equally weighted. The discrepancy measure between two datasets, ***x***^1^ and ***x***^2^ can be specified as,

$$\begin{array}{l} d(x^{1},x^{2})\equiv d(F(x^{1}),F(x^{2}))\\ \;=\frac{1}{8}\sum_{i=1}^{4}(1-\exp(-\rho (\mu_{i}^{1},\mu_{i}^{2},\sigma_{i}^{1},\sigma_{i}^{2})))\\ \;+\frac{1}{2}\sqrt{\frac{1}{16}\left(\sum_{i=1}^{4}{\left(ac_{i}^{1}-ac_{i}^{2}\right)}^{2}+\sum_{i=1}^{6}{\left(c_{i}^{1}-c_{i}^{2}\right)}^{2}+\sum_{i=1}^{6}{\left(cc_{i}^{1}-cc_{i}^{2}\right)}^{2}\right)}, \end{array}$$

where $\rho \left(\mu^{1},\mu^{2},\sigma^{1},\sigma^{2}\right)=\frac{1}{4}\log\left(\frac{1}{4}\left(\frac{\sigma^{1}}{\sigma^{2}}+\frac{\sigma^{2}}{\sigma^{1}}+2\right)\right)+\frac{1}{4}\left(\frac{{\left(\mu^{1}-\mu^{2}\right)}^{2}}{\sigma^{1}+\sigma^{2}}\right)$ is the Bhattacharya-coefficient (Bhattachayya, 1943) and 0 ≤ exp(−ρ(•)) ≤ 1. Further, we notice the value of the discrepancy measure is always bounded in the closed interval [0, 1].

#### Prior

We consider independent Uniform distributions for the parameters with a pre-specified range for each of them, *pAg* ~ *U*(5, 20), *pAd* ~ *U*(50, 150), *pT* ~ *U*(0.5*e* − 3, 3*e* − 3), *pF* ~ *U*(.1, 1.5), and *aT* ~ *U*(0, 10).

#### Perturbation kernel

To explore the parameter space of **θ** = (*pAg, pAd, pT, pF, aT*) ∈ [5, 20]×[50, 150] × [0.5*e*−3, 3*e*−3]×[.1, 1.5]×[0, 10], we consider a five-dimensional truncated multivariate Gaussian distribution as the perturbation kernel. SABC inference scheme centers the perturbation kernel at the sample it is perturbing and updates the variance-covariance matrix of the perturbation kernel based on the samples learned from the previous step.

### 3.4. Parameter estimation

Given experimentally collected platelet deposition dataset ***x*^0^**, our main interest is to estimate a value for **θ**. In decision theory, Bayes estimator minimizes posterior expected loss, $E_{p\left(\theta |x0\right)}\left(L\left(\theta ,•\right)|x^{0}\right)$ for an already chosen loss-function L. If we have *Z* samples ${\left(\theta_{i}\right)}_{i=1}^{Z}$ from the posterior distribution *p*(**θ**|***x*^0^**), the Bayes estimator can be approximated as,

(9) $$\begin{array}{r} \hat{\theta }=\underset{\theta }{\mathrm{argmin}}\frac{1}{M}\overset{M}{\underset{i=1}{\sum }}L\left(\theta_{i},\theta \right). \end{array}$$

As we consider the Euclidean loss-function $L\left(\theta ,\hat{\theta }\right)={\left(\theta -\hat{\theta }\right)}^{2}$ as the loss-function, the approximate Bayes-estimator can be shown to be $\hat{\theta }=E_{p\left(\theta |x^{0}\right)}\left(\theta \right)\approx \frac{1}{Z}\overset{Z}{\underset{i=1}{\sum }}\theta_{i}$.

## 4. Inference on experimental dataset

**Figure 5:**
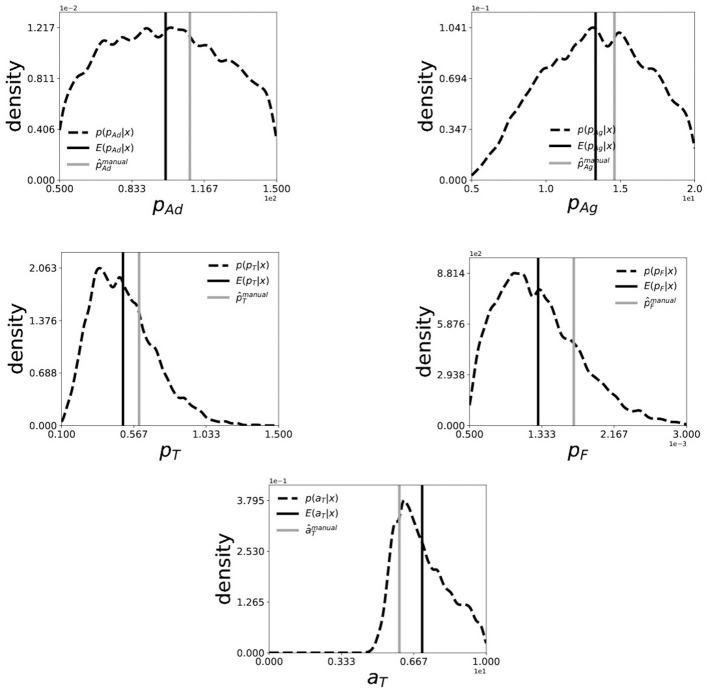
Marginal posterior distribution (black-dashed) and Bayes Estimate (back-solid) of (*pAd, pAg, pT, pF, aT*) for collective dataset generated from of seven patients. The smoothed marginal distribution is created by a Gaussian-kernel density estimator on 5000 i.i.d. samples drawn from the posterior distribution using SABC. The (gray-solid) line indicates the manually estimated values of the parameters in Chopard et al. (2017).

The performance of the inference scheme described in section 3 is reported here, for a collective dataset created from the experimental study of platelets deposition of seven blood-donors. The collective dataset was created by a simple average of $\left(Sagg-clust\left(t\right),{\mathbb{N}}_{agg-clust}\left(t\right),{\mathbb{N}}_{platelet}\left(t\right),{\mathbb{N}}_{act-platelet}\left(t\right)\right)$ over seven donors at each time-point *t*. In Figure 5, we show the Bayes estimate (black-solid) and the marginal posterior distribution (black-dashed) of each of the five parameters computed using 5000 samples drawn from the posterior distribution *p*(**θ**|***x*^0^**) using SABC. For comparison, we also plot the manually estimated values of the parameters (gray-solid) in Chopard et al. (2017). We notice that the Bayes estimates are in a close proximity of the manually estimated values of the parameters and also the manually estimated values observe a significantly high posterior probability. This shows that, through the means of ABC we can get an estimate or quantify uncertainty of the parameters in platelets deposition model which is as good as the manually estimated ones, if not better.

Next we do a Posterior predictive check to validate our model and inference scheme. The main goal here is to analyze the degree to which the experimental data deviate from the data generated from the inferred posterior distribution of the parameters. Hence we want to generate data from the model using parameters drawn from the posterior distribution. To do so, we first draw 100 parameter samples from the inferred approximate posterior distribution and simulate 100 data sets, each using a different parameter sample. We call this simulated dataset as the predicted dataset from our inferred posterior distribution and present the mean predicted dataset (blue-solid) compared with experimental dataset (black-solid) in Figure 6. Note that since we are dealing with the posterior distribution, we can also quantify uncertainty in our predictions. We plot the 1/4-th quantile, 3/4-th quantile (red-dashed), minimum and maximum (gray-dashed) of the predicted dataset at each timepoints to get a sense of uncertainty in the prediction. Here we see a very good agreement between the mean predicted dataset and the experimentally observed one, while the 1/4- and 3/4-th quantile of the prediction being very tight. This shows a very good prediction performance of the numerical model of platelet deposition and the proposed inference scheme.

**Figure 7:**
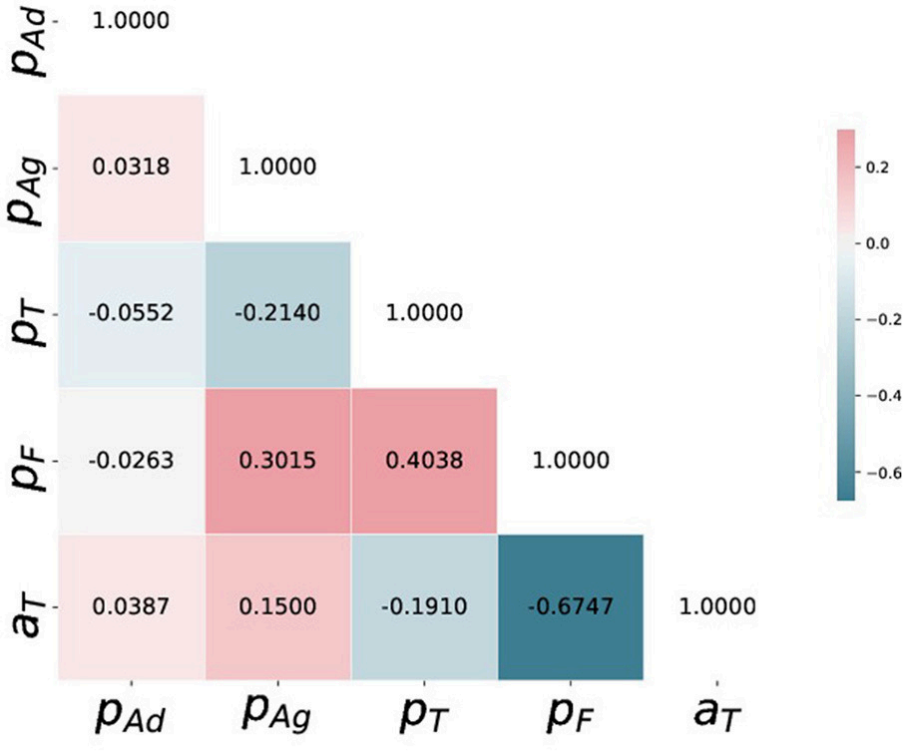
Posterior correlation matrix of (*pAd, pAg, pT, pF, aT*) computed from the 5000 i.i.d. samples drawn from the posterior distribution using SABC.

Additionally, to point the strength of having a posterior distribution for the parameters we compute and show the posterior correlation matrix between the five parameters in Figure 7, highlighting a strong negative correlation between (*pF, aT*), strong positive correlations between (*pF, pAg*) and (*pF, pT*). A detailed investigation of these correlation structure would be needed to understand them better, but generally they may point toward: (a) the stochastic nature of the considered model for platelet deposition and (b) the fact that the deposition process is an antagonistic or synergetic combination of the mechanisms proposed in the model.

Note finally that the posterior distribution being the joint probability distribution of the five parameters, we can also compute any higher-order moments, skewness etc. of the parameters for a detailed statistical investigation of the natural phenomenon.

## 5. Conclusions

Here, we have demonstrated that approximate Bayesian computation (ABC) can be used to automatically explore the parameter space of the numerical model simulating the deposition of platelets subject to a shear flow as proposed in Chopard et al. (2015); Chopard et al. (2017). We also notice the good agreement between the manually tuned parameters and the Bayes estimates, while saving us from subjectivity and a tedious manual tuning. This approach can be applied patient per patient, in a systematic way, without the bias of a human operator. In addition, the approach is computationally fast enough to provide results in an acceptable time for contributing to a new medical diagnosis, by giving clinical information that no other known method can provide. The clinical relevance of this approach is still to be explored and our next step will be to apply our approach at a personalized level, with a cohort of patients with known pathologies. The possibility of designing new platelet functionality test as proposed here is the result of combining different techniques: advanced microscopic observation techniques, bottom-up numerical modeling and simulations, recent data-science development and high performance computing (HPC).

Additionally, the ABC inference scheme provides us with a posterior distribution of the parameters given observed dataset, which is much more informative about the underlying process. The posterior correlations structure shown in Figure 7 may not have a direct biophysical interpretation, though it illustrates some sort of underlying and unexplored stochastic mechanism for further investigation. Finally we note that, although the manual estimates achieve a very high posterior probability, they are different from the Bayes estimates learned using ABC. The departure reflects a different estimation of the quality of the match between experimental observation and simulation results. As the ABC algorithms are dependent on the choice of the summary statistics and the discrepancy measures, the parameter uncertainty quantified by SABC in section 4 or the Bayes estimates computed are dependent on the assumptions in section 3.3 regarding their choice. Fortunately there are recent works on automatic choice of summary statistics and discrepancy measures in ABC setup (Gutmann et al., 2017), and incorporating some of these approaches in our inference scheme is a promising direction for future research in this area.

## Ethics statement

This study conforms with the Declaration of Helsinki and its protocol was approved by the Ethics Committee of CHU de Charleroi(comité déthique OM008). All volunteers gave their written informed consent.

## Data availability

The codes used to simulate the platelets deposition processes and to infer the process parameters from data can be downloaded from: https://github.com/eth-cscs/abcpy-models/tree/master/BiologicalScience/PlateletsDeposition.

## Author contributions

RD, BC, and AM design of the research. RD performed research. KZB and FD experimental data collection. RD and BC writing of the paper. AM, KZB, JL, FD, and AM contribution to the writing. BC and JL design and coding of the numerical forward simulation model.

### Conflict of interest statement

The authors declare that the research was conducted in the absence of any commercial or financial relationships that could be construed as a potential conflict of interest. The reviewer JB and handling Editor declared their shared affiliation
